# SPRi-Based Strategy to Identify Specific Biomarkers in Systemic Lupus Erythematosus, Rheumatoid Arthritis and Autoimmune Hepatitis

**DOI:** 10.1371/journal.pone.0084600

**Published:** 2013-12-20

**Authors:** Elvire Beleoken, Hervé Leh, Armelle Arnoux, Béatrice Ducot, Claude Nogues, Eleonora De Martin, Catherine Johanet, Didier Samuel, Mohammad Zahid Mustafa, Jean-Charles Duclos-Vallée, Malcolm Buckle, Eric Ballot

**Affiliations:** 1 Research Unit 785, Inserm, Villejuif, France; 2 Faculte de Medecine, University Paris-Sud, Villejuif, France; 3 Laboratoire de Biologie et de Pharmacologie Appliquée, ENS Cachan - CNRS, Cachan, France; 4 Unit 1018 - Centre for research in Epidemiology and Population Health, INSERM, Le Kremlin-Bicetre, France; 5 Unité de Recherche Clinique, AP-HP Hopital Bicetre, Le Kremlin-Bicetre, France; 6 Laboratoire Immunologie, AP-HP Hopital Saint-Antoine, Paris, France; 7 Faculté de Médecine UFR 967, Université Pierre et Marie Curie, Paris, France; 8 Centre Hépato-Biliaire, AP-HP Hôpital Paul Brousse, Villejuif, France; INSERM-Université Paris-Sud, France

## Abstract

**Background:**

Heterogeneous nuclear ribonucleoprotein (hnRNP) A2/B1 is a target for antinuclear autoantibodies in systemic Lupus erythematosus (SLE), rheumatoid arthritis (RA), and autoimmune hepatitis (AIH).

**Aim:**

To monitor molecular interactions between peptides spanning the entire sequence of hnRNP A2/B1 and sera from patients and healthy controls.

**Methods:**

Sera from 8 patients from each pathology and controls were passed across a surface plasmon resonance Imagery (SPRi) surface containing 39 overlapping peptides of 17 mers covering the human hnRNP B1. Interactions involving the immobilised peptides were followed in real time and dissociation rate constants k_off_ for each interaction were calculated.

**Results:**

Several significant interactions were observed: i) high stability (lower k_off_ values) between P_55-70_ and the AIH sera compared to controls (p= 0.003); ii) lower stability (higher k_off_ values) between P_118-133_ and P_262-277_ and SLE sera, P_145-160_ and RA sera compared to controls (p=0.006, p=0.002, p=0.007). The binding curves and k_off_ values observed after the formation of complexes with anti-IgM and anti-IgG antibodies and after nuclease treatment of the serum indicate that i) IgM isotypes are prevalent and ii) nucleic acids participate in the interaction between anti-hnRNAP B1 and P_55-70_ and also between controls and the peptides studied.

**Conclusions:**

These results indicate that P_55-70_ of hnRNP B1 is a potential biomarker for AIH in immunological tests and suggest the role of circulating nucleic acids, (eg miRNA), present or absent according to the autoimmune disorders and involved in antigen-antibody stability.

## Introduction

 Antinuclear autoantibodies against the heterogeneous nuclear ribonucleoprotein (hnRNP) A2/B1 are detected in autoimmune disorders, particularly several connective tissue diseases such as systemic lupus erythematosus (SLE), rheumatoid arthritis (RA) [1,2], but also in autoimmune hepatitis (AIH) [3].

**Figure 1:**
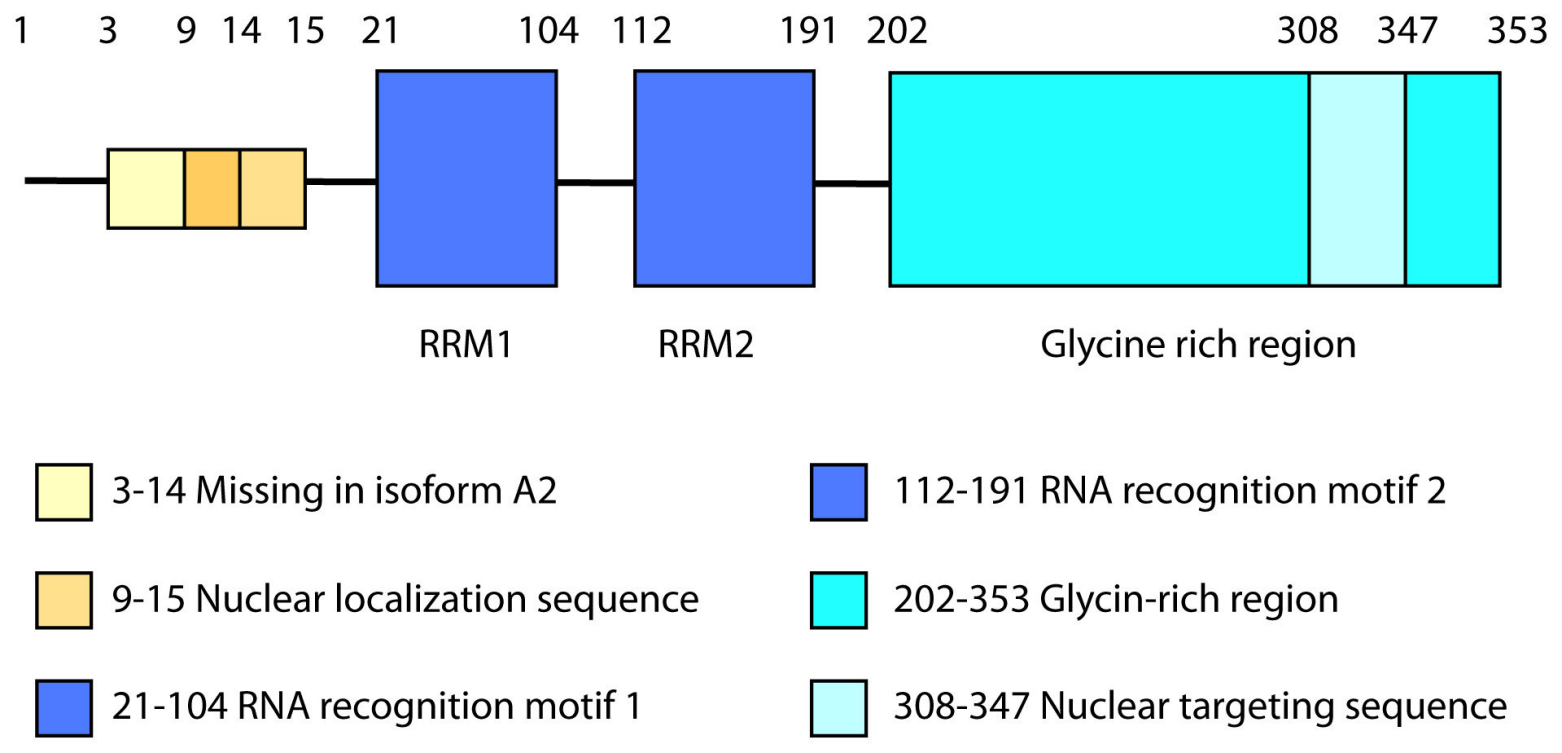
Major domains and regions in the complete isoform B1 of human hnRNP A2/B1 (access number P22626).

 HnRNP A2/B1 as part of the spliceosome, is involved in RNA processing and trafficking and in the splicing of many genes [4]. HnRNP A2 and B1 are two splicing variants of the same protein; the total B1 human sequence comprises 353 amino acids and the amino acids in position 3-14 are missing in the human isoform A2 [5]. The complete sequence contains two RNA recognition motif (RRM) domains (positions 21-104 and 112-191 in the N-terminal moiety), allowing their association in the nucleus with pre-mRNAs [5,6]. The C-terminal moiety is a glycine-rich region (position 202-353), which includes a nuclear target sequence (position 308-347) [7,8] (Figure 1). Using Enzyme-Linked ImmunoSorbent Assay (ELISA) and immunoblotting, a fine epitope mapping study involving 13 overlapping peptides spanning the RRMs of hnRNP A2 used as antigens, concluded that several peptides reacted with sera from patients with various rheumatic diseases [9].

In the ELISA approach, a positive signal reflects the quantity and the affinity of antibodies able to bind to antigens. However, two antibodies may share the same equilibrium dissociation constant K_D_, but have different rate constants for association (k_on_) and dissociation (k_off_) [10,11]. Also it is impossible to determine affinity data for unknown molecules of varying and undetermined concentrations in complex media such as sera. Since the dissociation rate constant is a unique and defining parameter characteristic of a given complex, we decided to make use of Surface Plasmon Resonance Imagery (SPRi) to explore the stability of the immune complex during dissociation. 

SPRi is a label free technique that uses prisms made of a high refractive index material with one surface coated with a thin layer of gold [12]. Biological material is covalently immobilised onto these surfaces and changes in concentration at the surface as macromolecules in solution interact with target molecules are followed in real time, allowing quantification of the interaction. Surfaces that are refractive to non-specific binding and which optimise presentation of immobilised ligands to the analyte in solution have recently been developed [13,14].. Under the conditions developed by Nogues et al [13,14], SPRi is ideal for high throughput experiments that screen complex physiological solutions for new biomarkers. 

 We demonstrate here the use of this innovative SPRi technology in autoimmunity studies in a peptide interaction display, using peptides spanning the entire sequence of hnRNP A2/B1 reacting with sera from patients with AIH and two systemic diseases, SLE and RA, compared to healthy controls. 

## Materials and Methods

### Sera studied

 Sera from patients and blood donors were collected (Laboratoire d’Immunologie, Hôpital Saint-Antoine, Paris, France), with approval of the Committee of the Biobanque du Centre hépatobiliaire, managed by the Biological Ressource Centre CRB Paris-Sud (http://www.chb.aphp.fr/rechercheClinique/biobanque/index.phtml). All subjects signed a written informed consent. Forty five sera from patients with autoimmune hepatitis as defined by the International Autoimmune Hepatitis Group [15] positive for antinuclear antibodies detected by indirect immunofluorescence on HEP2-cell monolayers and unfixed cryostat sections of rat liver (cut-off positivity ≥1:80), were tested by immunoblotting, using nuclear proteins resolved by 10% SDS-PAGE as antigen. Nuclear fractions from rat liver homogenate were obtained by centrifugation on sucrose density gradient as described elsewhere [3]. Sera were also collected from patients with pathologies known for high prevalence of anti-hnRNP A2/B1 autoantibodies, i.e., systemic lupus erythematosus SLE (n=30) and rheumatoid arthritis RA (n=57), diagnosed according to the criteria of the American College of Rheumatology. Sera from normal human blood donors (HD) (n=20) were included as negative controls. Sera that stained a double band at 36kDa on immunoblots were considered to be positive for anti-hnRNP A2/B1 autoantibodies, as described elsewhere [3]. hnRNP A2/B1 was thus recognized by 24 SLE sera (80%), 12 RA sera (21%), and by 22 AIH sera (48%). All the sera from blood donors were negative. Eight sera of each population were randomly used for further experiments against peptides, using SPRi technology. All sera were stored at -80°C until use. During SPRi experiments, the sera were diluted at 1:8000 in phosphate buffer saline (PBS).

### Peptide synthesis

 Thirty-nine 17 mer peptides with an overlap of seven amino acids were designed (Eurogentec, Angers, France). These peptides selectively covered the whole of the human hnRNP B1 isoform as reported in the SwissProt database under access number P22626 (Figure 1). They contained C-terminal thiol groups that permitted immobilisation on SPRi surfaces treated as described in [13]. Quality control was effective in 10% of peptides using the MALDI-TOF technique (Eurogentec, Angers, France). Grand Average of Hydropathicity (GRAVY) Index was calculated using ProtParam tool (http://web.expasy.org/protparam/) (Table S1). Before use, all peptides were suspended in distilled water (final concentration of 10mg/mL).

### SPRi

 Single surfaces containing multiple spots of all the peptides were constructed and interactions with components in whole sera from different patients were monitored and quantified in real time. 

Prisms were prepared essentially as previously described [13]. After 30 seconds of immersion in 1mM (11-mercaptoundecyl)tetra(ethylene glycol) (Sigma Aldrich, Saint-Quentin, France) solution in ethanol, the prisms were incubated in 1mM (11-mercaptoundecyl)tetra(ethylene glycol) 1-carboxylic acid ethanolic solution (Prochimia, Vallet, France) during 2 minutes. The surfaces were treated for 15 minutes with a mixture of 1-ethyl-3-(3-dimethylaminopropyl) carbodiimide hydrochloride (EDC) and N-hydroxysuccinimide (NHS) at final concentrations of 200mM and 50mM respectively (Amine coupling kit, GE Healthcare, Saclay, France). Prisms were then treated with a solution of 2-(2-pyridinyldithio) ethaneamine hydrochloride (PDEA) at a final concentration of 175mM (Thiol coupling kit, GE Healthcare) for 15 minutes. Un-reacted activated carboxyl groups on the surface were blocked by incubation with ethanolamine (1M at pH 8.5) for 10 minutes. The prisms were rinsed with distilled water and dried under pure argon gas. The peptides were then spotted onto the freshly pre-treated prism surface using a Hamilton Starlet® robot and a modified pin tool protocol that minimised contact of the pin tool with the gold coated surface. After 20 minutes of incubation in a humid chamber, the prisms were directly inserted into the SPRi apparatus and PBS buffer was immediately flowed across the surface at 25 µl/min.

The interaction of serum diluted 8000 times with PBS with the prism surfaces was examined by injecting diluted serum at 20µl/min in PBS across the surface at 22°C for 6 minutes. Following this injection phase the surfaces were continuously washed with PBS at 20µl/min for 45 minutes in order to follow dissociation of resulting complexes. In the case where anti-antibody was injected, anti-human IgG or anti-human IgM antibodies were injected at 1/800 dilution during the late dissociation phase. Anti human IgM antibodies were generously provided by Michael Tovey (LBPA, France), Anti human IgG antibodies (ab2410) were purchased from Abcam, France. The impact of nuclease treatment on the interactions between sera and peptides 7, 14, 17 and 30 was also examined by complementing the buffer with 10 units/ml of benzonase (Roche, France) and 10 units/ml RNase 1 (NEB, France). The sera were diluted 3200 times in the nuclease complemented PBS and incubated at room temperature 15 minutes before injection. 

### *k*_off_ determination

 Binding curves were obtained using the SPRi-Plex® (GenOptics, Orsay, France). Curves obtained from the interaction of sera and passive (PEG treated) surfaces containing no peptides were used to subtract from experiments involving surfaces with immobilised peptides. The use of an empty spot at least sets the bottom limit for the definition of non-specific binding. Effectively, there is no specific interaction between serum and the surface chemistry developed previously [12,13,14]. Furthermore, the aim of the study was not to quantify the amount of reactive material in the serum, but to define if something reacted specifically with target molecules on the surface. Since concentrations of reactive material in the sera were effectively unknowable, apparent dissociation rates (k_off_) were calculated at periods 25 to 35 minutes after the end of the injection phase using a simple exponential decay function to fit the dissociation phase using Origin® software, and in all cases fits were within the high confidence values of r > 0.99.

### Statistical analysis

 In order to compare the four groups of sera, values for the apparent k_off_ dissociation constant rates were analysed using the Kruskal Wallis test. Because of the multiplicity of statistical comparisons, the risk α was submitted to Dunn-Sidak’s correction, to reach a p-value threshold equal to 0.017.

## Results and Discussion

 HnRNPA2/B1 is an important protein in mRNA processing, export of RNA to cytoplasm and telomere biogenesis [16,17] and its expression is modified in a number of diseases [18]. hnRNP A2/B1 possesses many criteria that suggest it plays a role as an autoantigen [19], it is part of the spliceosome, it has alternative splicing events, it is able to bind to proteins and RNA and it is evolutionary conserved. It is thus a logical potential target for natural and disease associated autoantibodies. We report here the first SPRi-based strategy for the study of interactions between human sera and peptides that cover the hnRNP A2/B1 protein, which is one of the nuclear antigenic targets in SLE, RA and AIH. 

Since there exist common antigenic targets such as hnRNPA2/B1 in several autoimmune diseases, such as SLE, RA and AIH, it is therefore of interest to define specific markers to monitor these diseases. Since the identity and concentrations of molecules in sera that interact with the hnRNP A2/B1 protein are unknown, we decided to apply SPRi technology to use the measured apparent dissociation rate constants of complexes formed between peptides spanning the hnRNPA2/B1 molecule immobilised at a SPRi surface and putative target molecules in the sera of patients, and thus attempt to characterize the humoral response in different autoimmune diseases that involve hnRNPA2/B1 as autoantigen. 

**Figure 2:**
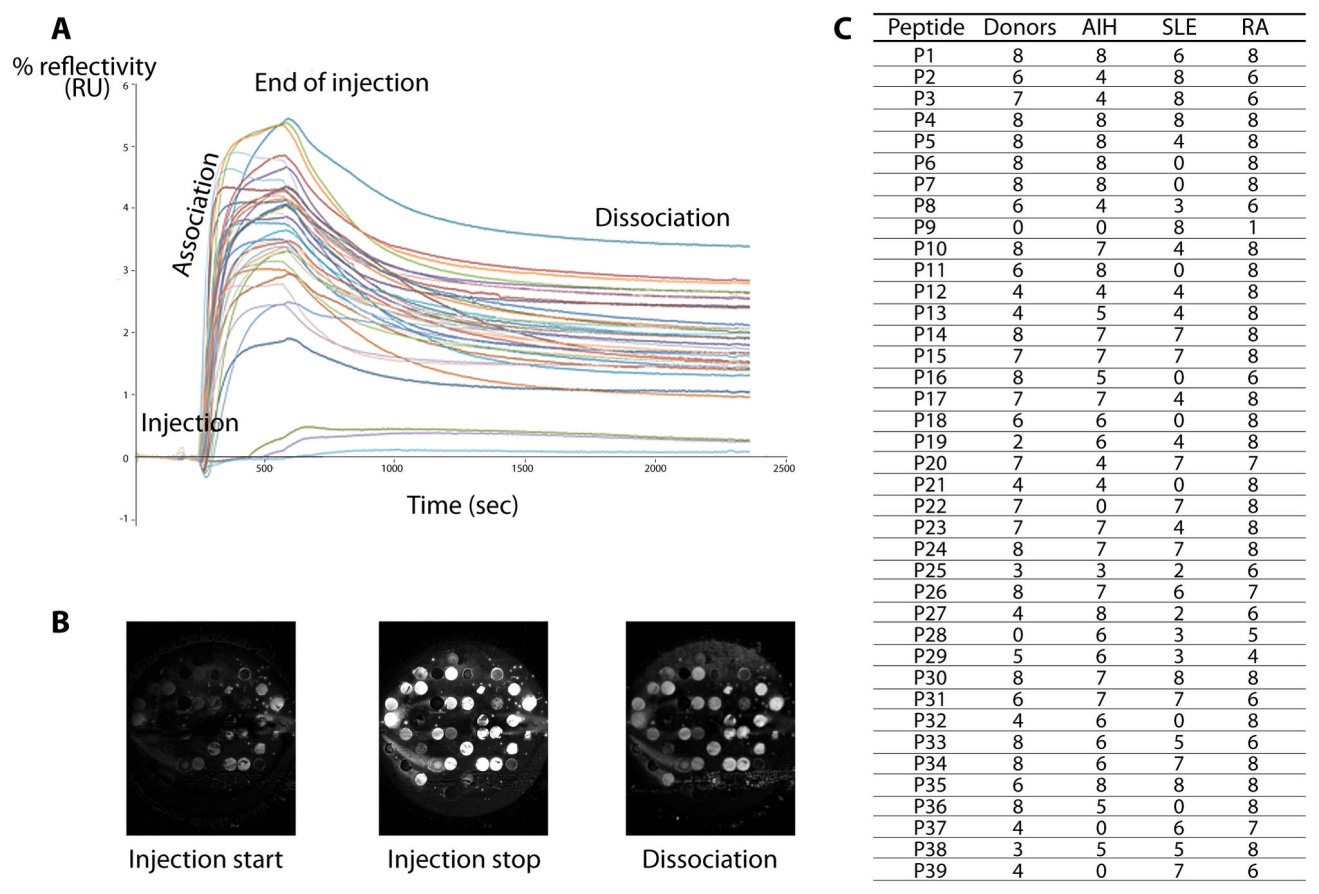
Interactions between peptides and sera. A. SPRi kinetic curves of serum and peptides immobilized on the surface of a prism. Changes in % reflectivity were measured as a function of time. Each curve shows binding to one of the peptides. B. SPRi difference images of the prism surface at different times. Peptide solutions at 10 mg/mL were spotted on the biochip surface. Difference images show the surface at the start of injection, during the injection and at the stop of injection. C. Number of effective interactions between the 39 peptides covering the sequence of hnRNP B1 and sera from healthy donors, autoimmune hepatitis (AIH), systemic lupus erytheamtosus (SLE), rheumatoid arthritis (RA) patients.

The C terminal Cys containing peptides were spotted on the same prism surface, allowing simultaneous interactions with each serum injected. Binding curves were obtained by reporting changes in % reflectivity as a function of time (Figures 2A and 2B). Each serum showed a different pattern of reactivity with the peptides. Only material from serum of patient RA7 was seen to bind to all the peptides. Conversely, material in all the sera interacted with peptide P4 (AA_28-43_). No material in serum from healthy donors bound to peptide P28 (AA_244-259_). Material from AIH sera did not bind to peptides P22 (AA_190-205_), P37 (AA_325-340_) and P39 (AA_340-353_) and material from SLE sera did not bind to peptides P6 (AA_46-61_), P7 (AA_55-70_), P11 (AA_91-106_), P16 (AA_136-151_), P18 (AA_154-169_), P21 (AA_181-96_), P32 (AA_280-295_) and P36 (AA_316-331_). Remarkably, only material present in all SLE sera, as well as in serum RA7, bound to peptide P9 (AA_73-88_) (Figure 2C). 

A number of 312 interactions (39 peptides x 8 sera) were expected in each group of sera but, only 74% of expected interactions in the group of HD, 69% in AIH sera and 56% in SLE sera were actually seen to bind to peptides, while the highest level of interactions (89%) was observed in the RA group. 

 Apparent dissociation rate constants k_off_ were calculated (Table S2) from the 1248 dissociation curves (312 interactions x 4 groups) (Figure 2C). Statistical analyses compared each group of patients with the donors on the one hand, and the groups of patients two by two on the other hand, detecting significant differences between groups (Tables 1, 2 and 3). 

**Table 1:** Comparison of k_off_ (s-1) between groups of sera.

**Peptides**	**Donors/AIH**	**Donors/SLE**	**Donors/RA**	**AIH/SLE**	**AIH/RA**	**SLE/RA**
**P6 (46-61)**	NS	NS	NS	NS	p=0.01	NS
**P7 (55-70)**	p=0.003	NS	NS	NS	p=0.01	NS
**P14 (118-133)**	NS	p=0.006	NS	NS	NS	p=0.006
**P17 (145-160)**	NS	NS	p=0.002	NS	NS	NS
**P20 (172-187)**	NS	NS	NS	NS	NS	p=0.004
**P30 (262-277)**	NS	p=0.007	NS	p=0.004	NS	NS
**P39 (340-353)**	NS	NS	NS	NS	NS	p=0.01

The dissociation rate constants k_off_ of sera from the three groups of patients were compared with those of healthy donors, using Kruskal-Wallis test with Dunn-Sidak’s correction (p-value ≤ 0.017). AIH, autoimmune hepatitis. RA, rheumatoid arthritis. SLE, systemic lupus erytematosus. NS, not statistically significant.

**Table 2:** koff (s-1) values of statistically significant comparisons of interactions between groups of sera from patients and donors, and peptides.

**Peptide**	**Peptide P7**		**Peptide P14**		**Peptide P30**		**Peptide P17**	
**p-value**	p=0.003		p=0.006		p=0.007		p=0.002	
**Group**	**AIH**	**Donors**	**SLE**	**Donors**	**SLE**	**Donors**	**RA**	**Donors**
**n**	8	8	7	8	8	8	8	7
k_off_ serum 1	0.00150	0.00192	0.00327	0.00272	0.00188	0.00359	0.00291	0.00161
k_off_ serum 2	0.00158	0.00286	0.00681	0.00285	0.00524	0.00322	0.00260	ND
k_off_ serum 3	0.00112	0.00232	0.00296	0.00253	0.00461	0.00252	0.00315	0.00213
k_off_ serum 4	0.00229	0.00352	0.00233	0.00125	0.00370	0.00122	0.00308	0.00250
k_off_ serum 5	0.00177	0.00328	ND	0.00153	0.00495	0.00121	0.00294	0.00218
k_off_ serum 6	0.00123	0.00210	0.00353	0.00224	0.00686	0.00255	0.00238	0.00158
k_off_ serum 7	0.00190	0.00217	0.00343	0.00148	0.00435	0.00295	0.00264	0.00151
k_off_ serum 8	0.00176	0.00201	0.00839	0.00239	0.00689	0.00328	0.00274	0.00192

k_off_ values in s^-1^; Kruskal Wallis test, Dunn-Sidak’s correction, p-value ≤ 0.017. ND, not determined; AIH autoimmune hepatitis; SLE systemic lupus erythematosus; RA Rheumatoid arthritis.

**Table 3:** k_off_ values (s^-1^) of statistically significant comparisons of interactions between groups of patient sera and peptides.

**Peptide**	**Peptide P30**		**Peptide P6**		**Peptide P7**		**Peptide P14**		**Peptide P20**		**Peptide P39**	
**p-value**	p=0.004		p=0.01		p=0.01		p=0.006		p=0.004		p=0.01	
**Group**	**AIH**	**SLE**	**AIH**	**RA**	**AIH**	**RA**	**SLE**	**RA**	**SLE**	**RA**	**SLE**	**RA**
**n**	7	8	8	8	8	8	7	8	7	7	7	6
k_off_ serum 1	0.00105	0.00188	0.00150	0.00296	0.00150	0.00296	0.00327	0.00166	0.00234	0.00160	0.00289	0.00118
k_off_ serum 2	ND	0.00524	0.00158	0.00172	0.00158	0.00172	0.00681	0.00248	0.00350	ND	0.00670	ND
k_off_ serum 3	0.00290	0.00461	0.00112	0.00239	0.00112	0.00239	0.00296	0.00165	0.00433	0.00192	0.00764	0.00160
k_off_ serum 4	0.00143	0.00370	0.00229	0.00281	0.00229	0.00281	0.00233	0.00254	0.00387	0.00215	0.00224	0.00102
k_off_ serum 5	0.00256	0.00495	0.00177	0.00171	0.00177	0.00171	ND	0.00254	0.00242	0.00228	0.00249	ND
k_off_ serum 6	0.00180	0.00686	0.00123	0.00250	0.00123	0.00250	0.00353	0.00176	0.00478	0.00194	ND	0.00188
k_off_ serum 7	0.00134	0.00435	0.00190	0.00251	0.00190	0.00251	0.00343	0.00266	0.00618	0.00246	0.00160	0.00230
k_off_ serum 8	0.00350	0.00689	0.00176	0.00253	0.00176	0.00253	0.00839	0.00170	ND	0.00155	0.00387	0.00047

k_off_ values in s^-1^ ; Kruskal Wallis test, Dunn-Sidak’s correction, p-value ≤ 0.017. ND, not determined; ND, not determined; AIH autoimmune hepatitis; SLE systemic lupus erythematosus; RA Rheumatoid arthritis.

**Figure 3:**
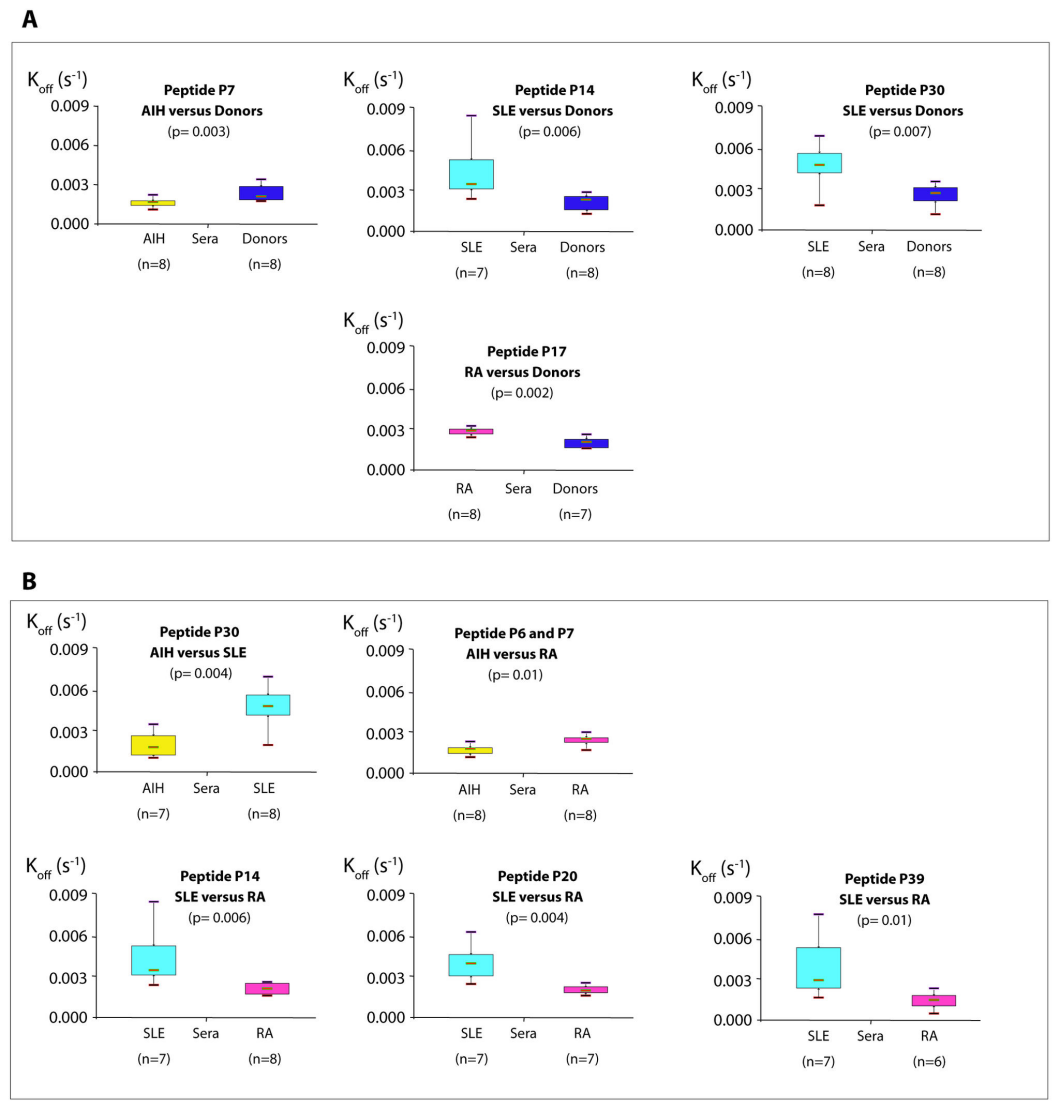
Comparisons of apparent k_off_ (s^-1^)values. A. Between groups of patients and donors. Each group of patients was compared with a group of healthy donors. Complexes between AIH (autoimmune hepatitis) sera and peptide P7 (AA_55-70_) were more stable than with healthy control sera. Conversely, complexes between donor sera and peptides P14 (AA_118-133_) and P30 (AA_262-277_) were more stable than with SLE (systemic lupus erytheamtosus) sera. The same applied to peptide P17 (AA_145-160_) and RA (rheumatoid arthritis) sera. B. Between groups of patients. Complexes between AIH sera, peptides P6 (AA_46-61_) /P7 (AA_55-70_) and P30 (AA_262-277_) were more stable than those formed respectively by RA and SLE sera. SLE sera also formed complexes less stable than RA sera with peptides P14 (AA_118-133_), P20 (AA_172-187_), P39 (AA_340-353_).

Figure 3 summarizes the results of the different comparisons. The complexes formed by the sera of AIH patients with peptide P7 were more stable than with sera from HD. Indeed, we observed a significant interaction between peptide P7 and the AIH sera, with a lower k_off_ compared to the controls (p= 0.003) (Table 2), suggesting a high stability of the complexes generated by AIH sera compared to those generated with control sera (Figure 3A). This result suggests that peptide P7 (AA_55-70_) could be a specific biomarker for AIH in human sera. 

Surprisingly, complexes formed with peptides P14 (AA_118-133_) and P30 (AA_262-277_) by SLE sera were less stable with a significantly higher k_off_ than those formed by HD sera (p=0.006 and p=0.007 respectively) (Figure 3A, Table 2). This also applies to the interactions between peptide P17 (AA_145-160_) and sera from RA patients and HD, and with a significant difference in k_off_ (p=0.002) (Figure 3A, Table 2). Interactions between peptide P30 (AA_262-277_) and AIH sera were found to be more stable than with SLE sera where significant larger k_off_ values were found than observed for AIH sera (p=0.004) (Figure 3B, Table 3). Similarly, k_off_ values for the reactions between AIH sera and peptides P6 (AA_46-61_) and P7 (AA_55-70_) were lower than those calculated with RA sera (p=0.01 for both) (Figure 3B, Table 3). Dissociation rate constants were also lower for interactions between peptides P14 (AA_118-133_), P20 (AA_172-187_), P39 (AA_340-353_) and RA sera than with SLE sera (p=0.006, p=0.004 and p=0.01 respectively for the three peptides), suggesting that the complexes involving these peptides and SLE sera were less stable than those involving the same peptides and components from RA sera (Figure 3B, Table 3). 

Peptides P14 (AA_118-133_) and P17 (AA_145-160_) belong to the RRM2 region. This observation therefore agrees with previous studies that used bacterially expressed fragments and showed that the major epitopes of hnRNPA2 detected by RA sera and SLE sera are located in the RRM2 domain [20]. Moreover, Schett et al. [9] reported that anti hnRNP A2 antibodies in SLE sera were predominantly directed to three major antigenic regions matching with sequences corresponding to AA_47-62_, AA_102-128_, AA_167-187_ in B1 isoform, and also with sequence AA_62-82_, the latter with a low reactivity. The last three sequences overlapped in our experiments with peptides P14 (AA_118-133_), P20 (AA_172-187_) and P9 (AA_73-88_). 

The case of SLE sera is of particular interest since they were the only ones (except for serum RA7) that bound to peptide P9 (AA_73-88_). With respect to peptide P30 (AA_262-277_) and P39 (AA_340-353_) that derives from the glycine-rich region, Sun et al reported [21] that in SLE some autoantibodies to double-stranded DNA cross-react with the arginine-glycine rich domain. Furthermore, the major epitope recognized by SLE sera is reported to correspond to region AA_167-187_ of isoform B1 of hnRNP molecule.

One explanation of these significantly lower k_off_ values may be inherent to the technique. The ELISA technology often used in epitope mapping is an endpoint assay, in which a positive signal reflects affinity at equilibrium (or at some steady state) given by an apparent dissociation equilibrium binding constant K_D_, (also defined as the ratio of the dissociation rate constant k_off_ to the association constant k_on_). Two antibodies may have the same dissociation binding constant K_D_, but differ in their respective on and off kinetic constants [10,11]. SPRi by measuring apparent on and off rates allows calculation of kinetic constants and estimation of the apparent dissociation binding constant without necessarily requiring an end point or steady-state/equilibrium, in contrast to ELISA which *a priori* provides little information of the kinetics and especially of the dissociation rate constant unless used in competition assays.

In this study, the nature of the reacting species involved was not known. We also have no way of knowing the concentration of the reacting species in the sera. For these reasons, it is impossible to use the law of mass action to quantify the association part of the binding curves of the sensorgram, this is why we examine only the dissociation parts of the sensorgram taking advantage of the fact that the kinetics of dissociation are independent of the concentration of the reactants. The apparent kinetic constant dissociation thus reflects the stability of complexes formed during the interaction of the peptides with sera.

**Figure 4:**
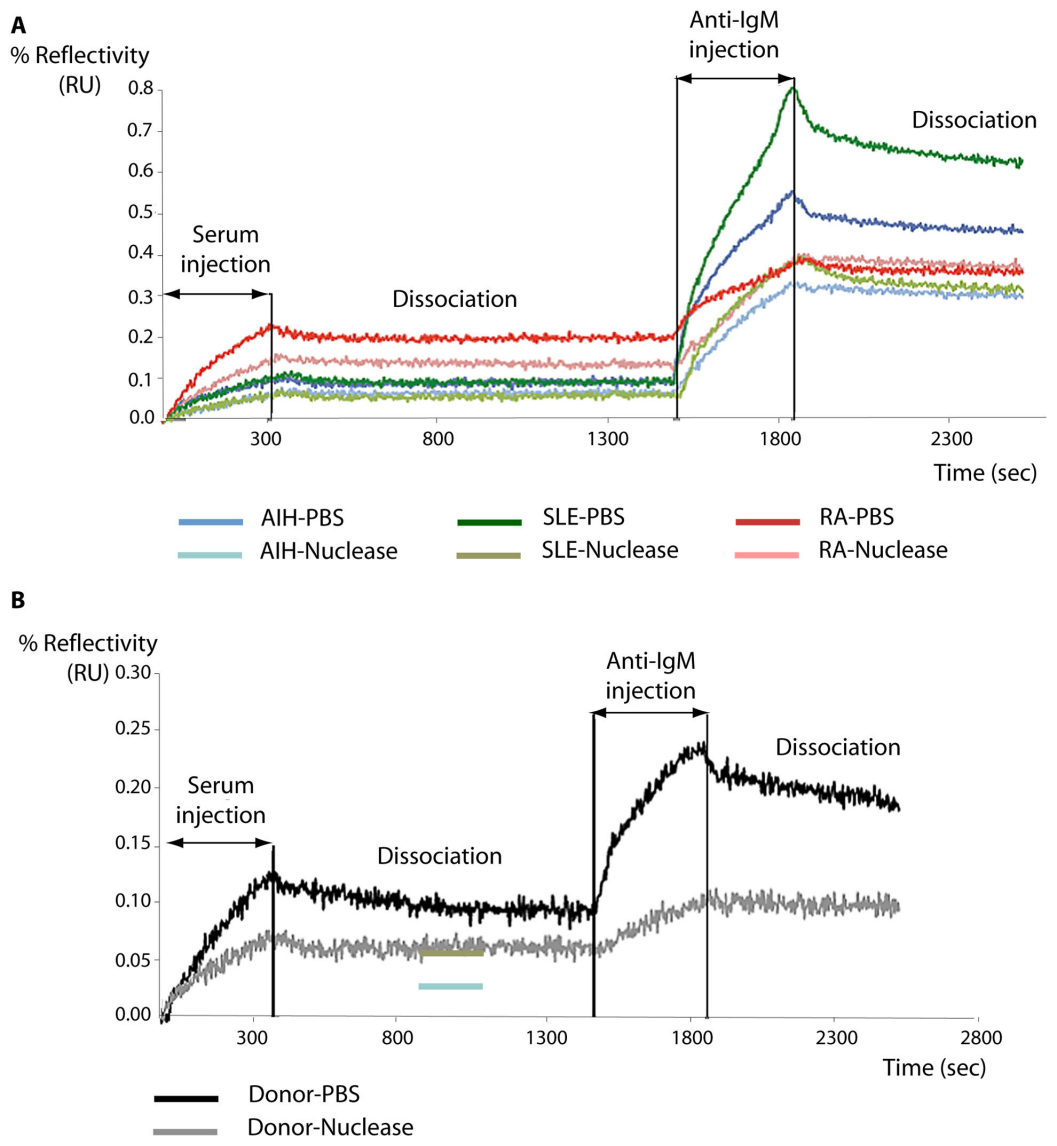
Anti IgM binding to material from serum retained at immobilised peptide P7 in the presence and absence of nucleases. Sera were passed across immobilised peptides and the resulting complexes were allowed to dissociate so that only the most stable complexes remained. Anti-IgM was then flowed across the surfaces. The experiment was repeated after pre-treatment with, and in the presence of, nucleases as described in Materials and Methods. A. P7 Binding curves for AIH (autoimmune hepatitis), RA (rheumatoid arthritis) and SLE (systemic lupus erytheamtosus) sera binding to immobilised P7 and subsequent binding of anti-IgM antibodies in the presence or absence of nucleases. B. P7 Binding curves for donor sera binding to immobilised P7 and subsequent binding of anti-IgM antibodies in the presence or absence of nucleases.

The exact nature of the molecule in human sera that interacts with the peptides on the biochip is unknown but the differences in reactivity with P14 (AA_118-133_), P30 (AA_262-277_) and P17 (AA_145-160_) on the one hand, and with P7 (AA_55-70_) on the other hand are suggestive of a conformational difference between both groups with low and high apparent k_off_ values. We suspected that perhaps IgG or IgM moieties might be involved in, or mediate interactions with, these peptides. We therefore reformed complexes by passing sera from patients and donors across surfaces containing the peptides then challenged these surfaces with anti-IgG and anti-IgM antibodies. Whereas no reaction was seen between complexes at the surface and anti-IgG molecules, as seen in Figure 4, anti IgM molecules strongly reacted (for clarity we show only peptide P7 (AA_55-70_), although all material selectively retained at all peptides cross-reacted with anti IgM antibodies). In the case of peptide P7 (AA_55-70_), one could then postulate that certain IgM molecules are present and that they recognize the specific epitope afforded by peptide P7 (AA_55-70_) to form stable complexes. The observation of the binding curves and the binding kinetics obtained in real time after dissociation of complexes with anti-antibodies (Figure 4) leads to the conclusion that most autoantibodies that react with P7 (AA_55-70_) belong to the M isotype. This distribution is compatible with the presence of natural antibodies detected in autoimmune diseases and in healthy subjects, for whom IgM titers in sera are high [22]. 

How then to explain the decreased stability of complexes formed between other peptides and IgM especially in view of the observation that all the peptides reacted with IgM? 

 A first explanation is that the natural antibodies exhibit a plastic paratope that has not undergone somatic mutations involved in affinity maturation. These antibodies have a low affinity and one could hypothesize that the complexes formed are of a low affinity. 

An alternative hypothesis is that interactions with the peptides and IgM molecules are mediated by a third moiety. It has been reported that anti-hnRNPA2 antibodies from SLE and RA patients are able to inhibit the binding of RNA [20] and that their association with nucleic acids [23] mediates the antigenic properties of hnRNP. It is also of interest that practically all the peptides that we identified as reacting with IgMs in sera, derived from the RRM regions of hnRNP. We therefore suspected that nucleic acids might be involved in some way with the formation of stable complexes between the peptides and IgM molecules in the sera. We therefore treated the sera with nuclease in order to eliminate as thoroughly as possible any poly nucleic acids that may be present. As seen in Figure 4A this treatment severely reduced the amount of IgM binding to P7 from AIH, PR and SLE patients. Significantly, nuclease treatment also reduced the binding of IgM of donor patient sera to peptide P7 (Figure 4B). Thus some form of poly-nucleic acid present in the sera is clearly implicated in the formation of complexes between IgM molecules and select peptides from the hnRNP A2/B1 protein. 

Interactions of immune complexes including RNA with TLR7 have been proposed to regulate the auto reactive B cell response [24]. We suggest the existence of a tertiary complex between antibodies and epitopes resulting in interactions between the RRM2 domain and other proteins or nucleic acids. Consequently, less stable complexes would be formed if one component were missing, with a non-optimal fit between antibodies and epitope. The relatively widely dispersed values of k_off_ observed with SLE patients may be due to a broader range of potential interactions leading to a wider spectrum of relatively weaker complexes being formed. We postulate that RNA molecules may be suitable candidates because of their abundance in human sera. In other words, RNA molecules, in combination with the peptides tested, constitute the epitopic area. Likely candidates for this role are miRNAs that are non-coding RNAs of about 21 nucleotides implicated in post-transcriptional regulation of gene expression. miRNA’s play an important role in the regulation of immune functions [25] and are potentially involved in the pathogenesis of autoimmune diseases, specifically RA and SLE [26]. To our knowledge, miRNAs have not been reported in AIH. Aberrant expression of down-regulated miRNAs reported in SLE and RA [27,28] suggested a crucial role of particular microRNAs in the establishment of B cell tolerance and the prevention of auto reactive antibodies.

This study using an SPRi strategy, identified a potential biomarker in sera from AIH patients, compared to SLE and RA patients, and suggested the implication of nucleic acid "facilitators" in the recognition of epitopes determinant for autoimmune diseases. The technology is thus demonstrably valid for the identification and characterization of biomarkers in autoimmune diseases. 

## Supporting Information

Table S1 **List of thirty-nine 17 mer synthtetic peptides covered the whole of the human hnRNP B1 isoform (P22626).**
(DOCX)

Table S2 **Apparent dissociation rate constant k_off_ (s^-1^) calculated from the 1248 dissociation curves.**
(XLS)
