# Supplementary material for: SPRi-Based Strategy to Identify Specific Biomarkers in Systemic Lupus Erythematosus, Rheumatoid Arthritis and Autoimmune Hepatitis
Source: PLoS One. 2013 Dec 20;8(12):e84600. doi: 10.1371/journal.pone.0084600 (PMC3869893; doi:10.1371/journal.pone.0084600)
Supplement: Table S1 — List of thirty-nine 17 mer synthtetic peptides covered the whole of the human hnRNP B1 isoform (P22626). (DOCX) [file pone.0084600.s001.docx]

**Table S1.** List of synthtetic peptides covered the whole of the human hnRNP B1 isoform (P22626).

| **PEPTIDE** | **Amino acid** | **Peptide sequence (N-term, C-term)** | **GRAVY index** |
| --- | --- | --- | --- |
| P1 | 1-16 | MEKTLETVPLERKKREC | -1.265 |
| P2 | 10-25 | LERKKREKEQFRKLFIC | -1.347 |
| P3 | 19-34 | QFRKLFIGGLSFETTEC | 0.065 |
| P4 | 28-43 | LSFETTEESLRNYYEQC | -1.071 |
| P5 | 37-52 | LRNYYEQWGKLTDCVVC | -0.353 |
| P6 | 46-61 | KLTDCVVMRDPASKRSC | -0.4 |
| P7 | 55-70 | DPASKRSRGFGFVTFSC | -0.294 |
| P8 | 64-79 | FGFVTFSSMAEVDAAMC | 1.106 |
| P9 | 73-88 | AEVDAAMAARPHSIDGC | 0.065 |
| P10 | 82-97 | RPHSIDGRVVEPKRAVC | -0.624 |
| P11 | 91-106 | VEPKRAVAREESGKPGC | -1.035 |
| P12 | 100-115 | EESGKPGAHVTVKKLFC | -0.382 |
| P13 | 109-124 | VTVKKLFVGGIKEDTEC | 0.106 |
| P14 | 118-133 | GIKEDTEEHHLRDYFEC | -1.447 |
| P15 | 127-142 | HLRDYFEEYGKIDTIEC | -0.865 |
| P16 | 136-151 | GKIDTIEIITDRQSGKC | -0.518 |
| P17 | 145-160 | TDRQSGKKRGFGFVTFC | -0.712 |
| P18 | 154-169 | GFGFVTFDDHDPVDKIC | -0.024 |
| P19 | 163-178 | HDPVDKIVLQKYHTINC | -0.476 |
| P20 | 172-187 | QKYHTINGHNAEVRKAC | -1.194 |
| P21 | 181-196 | NAEVRKALSRQEMQEVC | -0.853 |
| P22 | 190-205 | RQEMQEVQSSRSGRGGC | -1.529 |
| P23 | 199-214 | SRSGRGGNFGFGDSRGC | -1.012 |
| P24 | 208-223 | GFGDSRGGGGNFGPGPC | -0.624 |
| P25 | 217-232 | GNFGPGPGSNFRGGSDC | -0.829 |
| P26 | 226-241 | NFRGGSDGYGSGRGFGC | -0.8 |
| P27 | 235-250 | GSGRGFGDGYNGYGGGC | -0.776 |
| P28 | 244-259 | YNGYGGGPGGGNFGGSC | -0.606 |
| P29 | 253-268 | GGNFGGSPGYGGGRGGC | -0.612 |
| P30 | 262-277 | YGGGRGGYGGGGPGYGC | -0.7 |
| P31 | 271-286 | GGGPGYGNQGGGYGGGC | -0.771 |
| P32 | 280-295 | GGGYGGGYDNYGGGNYC | -0.988 |
| P33 | 289-304 | NYGGGNYGSGNYNDFGC | -1.135 |
| P34 | 298-313 | GNYNDFGNYNQQPSNYC | -1.753 |
| P35 | 307-322 | NQQPSNYGPMKSGNFGC | -1.265 |
| P36 | 316-331 | MKSGNFGGSRNMGGPYC | -0.753 |
| P37 | 325-340 | RNMGGPYGGGNYGPGGC | -0.947 |
| P38 | 334-349 | GNYGPGGSGGYGGC | -0.657 |
| P39 | 340-353 | GSGGYGGRSRYC | -1.058 |

GRAVY index (Grand Average Hydropathicity Index): if >0, hydrophobic protein

if< 0, hydrophilic protein
